# A Method of Treating Putrescent Root-Canals and Opening Fine and Constricted Ones for Sterilization

**Published:** 1901-02-15

**Authors:** F. T. Hays

**Affiliations:** Chicago, Ills.


### A METHOD OF TREATING PUTRESCENT ROOT-CANALS AND OPENING FINE AND CONSTRICTED ONES FOR STERILIZATION.

BY F. T. HAYS, D.D.S., CHICAGO, ILLS.

I desire to report my experience with a method which I have found both rapid and efficient. Driven to exasperation by the use of sulfuric acid for the above named purpose, I experimented with a view to finding something which would not corrode every instrument with which it came in contact, and would still act with sufficient rapidity in such cases and insure certainty and expedition.

I have found that nitro-hydrochloric acid, or "aqua regia," does not corrode a steel broach further than to cause a thin coating on the surface, which can be easily removed by a cuttlefish disk. And the same broach may be used to carry the medicament to canals, to be left there until termination of the treatment, without any fear that the broach may have been weakened and may break off, as is the case when $H_2SO_4$ is so used. In the treatment of putrescent root canals with aqua regia an effervescence is produced almost equal to that caused by hydrogen dioxid. This effervescence assists in carrying out and into the pulp chamber much of the debris lodged in the canals. At the same time an elimination of free chlorin takes place, which helps to further sterilize the canals and render innocuous any putrescent matter that may remain therein, at the same time bleaching the tissue and leaving it nice and white after thoroughly drying out. The bleaching effect of the gas enables the operator to better see the canals and more easily treat them.

I have many times opened into a dead pulp, left the canal open for a few days to drain and that soreness might subside, and at the next sitting subjected the tooth to the treatment as outlined, sealed it up tightly and left it for one week without any inconvenience to the patient. Rarely do I have to treat a tooth by this method more than once thereafter.

I find that I effect a saving of much time to both the patient and myself, and I have cleaner and better canals in which to insert a filling than results from any method heretofore described with which I am acquainted.

As for its use in fine and constricted canals, my advice is to try it and be convinced of the time saved over the old method; besides, one does not require a special broach, as is the case with $H_2SO_4$.

After using any acid in canals, the residu of acid should be carefully neutralized and the canals dried thoroughly before putting a dressing in them, as this avoids the dilution of medicaments used as a dressing and assists the tissue at the apex to return to normal condition because of aseptic surroundings and absence of irritating fluids to irritate the apical tissue.

My method of procedure is this: Thoroughly exclude the surrounding tissue, preferably by means of the rubber dam; if this is not convenient, have the assistant protect the parts with some absorbent material, thereby excluding saliva and its inevitable consequence, the carrying out over the walls and surfaces of the teeth some of the agent with which you are working, which should always be avoided when working with a corrosive. Then with a fine, smooth broach, around which have been wound a few threads of cotton, carefully and gradually work the medicament toward the apical end. Use as fine a broach and as few threads of cotton as consistent with the needs of the case, as a broach that tightly fills up the canal acts as a piston and many times forces septic material through the apical foramen and thereby excludes the possibility of its sterilization by the method outlined; soreness and discomfort, moreover, are sure to follow.

As to any harmful disintegration of tooth structure, I have never been able to demonstrate that, and I have watched this matter closely as a clinician before reporting. But I do not get the material on the tissue except at the opening of the canal, as the smallest possible amount is quite sufficient; and by due care it can be kept away from where it may do possible injury.

The same care and certainty is often difficult in the use of sulfuric acid "in any degree of concentration," as with it you can not use the fine broach and cotton medium to carefully place in a fine canal; hence the almost certainty of its spreading over the floor of the pulp chamber, there to remain until neutralized at the termination of the acid treatment, and to do more injury than need be the case when carefully following the technique of this method.

Again, I have many times used the method here advocated in the canals of anterior teeth that I intended reaming out for a Logan pin, and have tried to differentiate, in the cutting of the tissue immediately around the canal, that portion in which the acid came in actual contact, but could detect no difference so far as my sense of touch could specialize.

I have not as yet made any extensive laboratory experiments, but have used this material freely in roots of freshly extracted teeth, carefully neutralized and dried out, and on splitting open the canals have found them pure and clean, the tissue hard and dense, with noxious odors well dissipated. If by chance the agent should come in contact with the tissue in the apical region, I have found nothing more than a persistent tendency to the formation of serum, but have had no trouble from a root filled in this condition if the canals are perfectly aseptic. Of corrse I should treat such a case more often than suggested, and try to overcome the tendency. But we sometimes meet such cases from some other cause, and find it quite impossible to check the serumal flow when we are called upon to fill the roots and so terminate the treatment.

As to the medicaments to be used for canal dressing, they should be soothing and antiseptic. I prefer oil of cloves, because it produces slight anesthesia of the parts and aborts the condition described by patients as a drawing sensation which follows the use of astringents, and many times lasts for ten or twelve hours. The operator should be thoroughly familiar with the action of whatever canal medicaments he uses, and the conditions in which he should find the canal when he deems the treatment finished and proceeds to fill the canal. Much suffering can be caused by the use of powerful deodorants, which mislead the operator in terminating the treatment too quickly. I use oil of cloves for final dressings because the odor is easily dissipated in the presence of noxious gases and I am not misled, as might be the case with such medicines as thymol and oil of wintergreen. Oil of cassia I have no use for under a sealed dressing.

I offer the foregoing suggestions hoping they may be of value to others under perplexing circumstances. I have no hesitation in recommending the method to the profession. If they will remember the technique and neutralize the agent when through with it, I should expect less injury from it than from any similar method and much benefit to patients who are the unfortunate possessors of fine and constricted canals.—*Dental Cosmos.*

